# Investigation of *Anaplasma* Species with Veterinary and Public Health Significance in Sheep and Goats

**DOI:** 10.1007/s11686-025-01056-5

**Published:** 2025-05-27

**Authors:** Mustafa Karatepe, Münir Aktaş, Bilge Karatepe, Sezayi Özübek

**Affiliations:** 1 Faculty of Science, Department of Biotechnology, Niğde Ömer Halisdemir University, 51240 Niğde, Türkiye; 2 Faculty of Veterinary, Department of Parasitology, Fırat University, 23200 Elazığ, Türkiye

**Keywords:** Sheep, Goat, *Anaplasma*, Tick

## Abstract

**Purpose:**

This study was carried out to investigate *Anaplasma* important for veterinary and public health in sheep and goats in Niğde province in Türkiye by using molecular methods.

**Methods:**

Blood samples were taken from randomly selected 690 animals (520 sheep and 170 goats), which were between 1 and 10 years old and from different study sites in Niğde by using the vacutainer tubes containing EDTA. After the genomic DNA extractions samples, the *Anaplasma* spp. 16S rRNA genes were amplified by PCR. Species-specific polymerase chain reaction (PCR) assays were performed on positive samples for the presence of *A. bovis*, *A. capra*, *A. ovis, A. platys*-like, and *A. phagocytophilum*. At the same time, the animals were tested for ixodid tick infestation and collected ticks were examined for identification under the stereo-microscope.

**Results:**

The results of PCR analysis show that the overall *A. ovis* prevalence was 63.3% (437/690) in small ruminants sampled. A total of 361 sheep (69.4%) and 76 goats (44.7%) were found to be infected with *A. ovis*, whereas no positivity was detected for *A. bovis*, *A. capra*, *A. platys*-like, and *A. phagocytophilum. Anaplasma ovis* positivity was observed at the highest percent in May (%74.6) while the lowest in June (%52.4). In total, 1361 ticks (579♀, 782♂) were collected from sheep and goats in Niğde. Ticks were identified as *Rhipicephalus bursa* (383, 28.1%), *R. turanicus* (607, 44.6%), *Hyalomma marginatum* (7, 0.5%), *Hy. excavatum* (247, 18.1%), *Hy. anatolicum* (23, 1.7%), *Haemophsalis parva* (21, 1.5%), *Hae. punctata* (7, 0.5%), *Hae. sulcata* (40, 2.9%) and *Dermacentor marginatus* (26, 1.9%).

**Conclusion:**

The present study reports a high prevalence of *A. ovis* 63.3% (437/690) in sheep and goats in Niğde province.

## Introduction

Anaplasmosis is caused by organisms of the genus *Anaplasma*, which are gram negative bacterium and transmitted to cattle, sheep, goats, wild ruminants, carnivores and humans by ticks [1, 2]. *Anaplasma ovis* and *A. phagocytophilum* species have been reported to cause clinical and subclinical infections in sheep and goats [2]. *Anaplasma ovis* usually causes mild disease in ruminants. It has been observed that it causes clinical infections in sheep and goats, especially in the presence of various factors (co-infections or stressful situations). *Anaplasma ovis* infections have been reported to be endemic throughout the world, including Europe, China and the United States [3]. *Anaplasma phagocytophilum*, which causes the infection called tick-borne fever in ruminants, causes important infections in ruminants, equines, canines, and humans. Tick-borne fever can cause direct (lamb death) and indirect loss (growth reduction) in sheep, moreover, up to 30% lamb deaths have been reported due to *A. phagocytophilum* [4]. In 2015, a new species named *A. capra* was discovered in goats and ticks in China. In addition, this species has been reported to cause infection in humans [5]. *Anaplasma bovis* causing monocytic anaplasmosis in cattle has been reported in sheep and goats [6]. Similarly, *A. platys* causing cyclic thrombocytopenia in dogs, it has been reported in sheep and goats as *A. platys*-like [6, 7]. The importance of *A. bovis* and *A. platys*-like in sheep and goats is unknown. This study aimed to investigate *Anaplasma* species which have veterinary and public health importance in sheep and goats in Niğde, Türkiye.

## Materials and Methods

### Study Area and Sample Collection

**Fig. 1:**
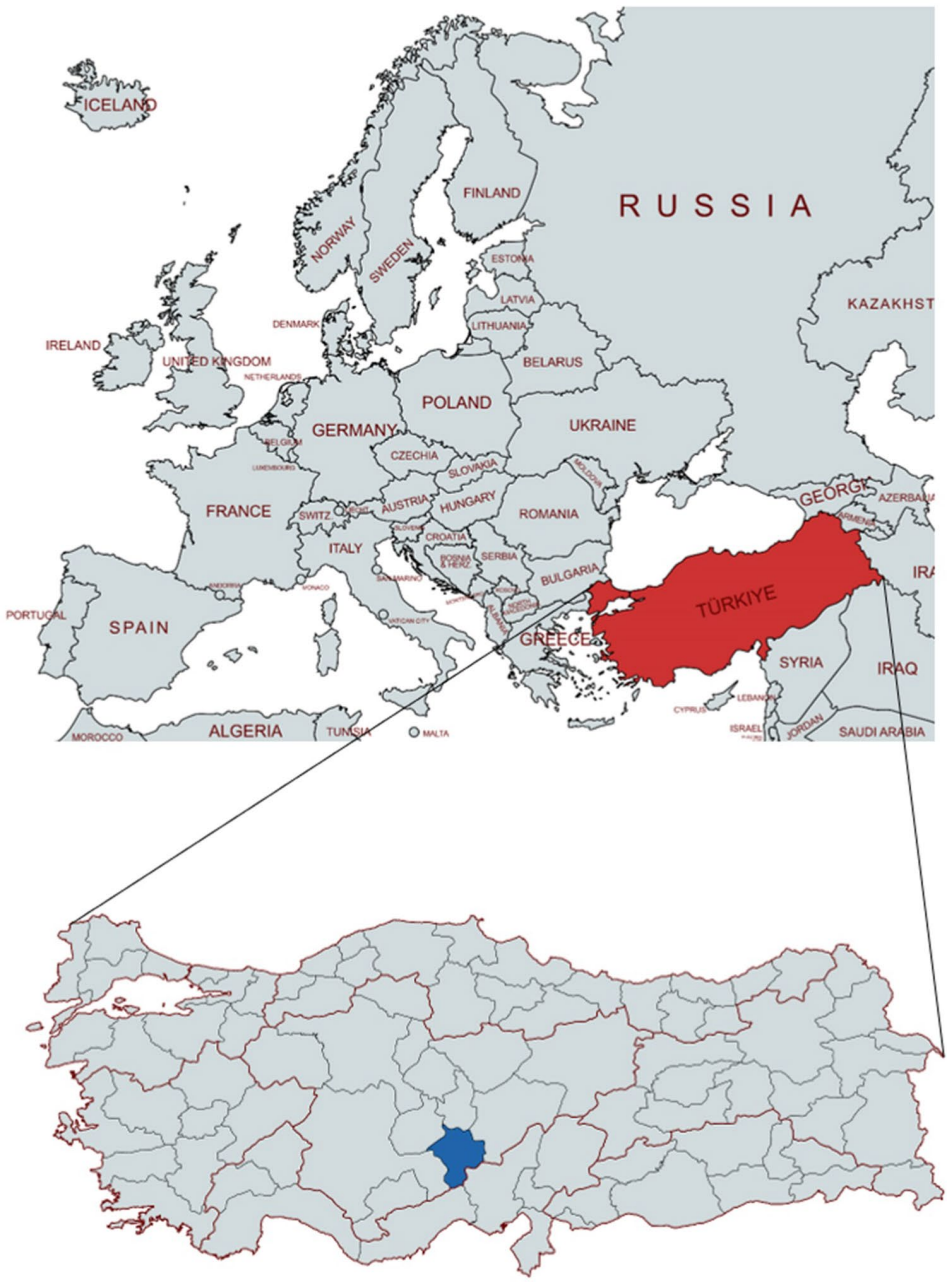
Geographic location of the study area. The map displays Türkiye in red, situated at the crossroads of southeastern Europe and western Asia. The Niğde province, where the study was conducted, is highlighted in blue in the inset map. The figure was created using mapchart.net

Blood samples were collected from apparently healthy sheep (n = 520) and goats (n = 170) belonging to 28 flocks of Niğde province, in Central Anatolia of Türkiye (with an altitude of 1240 m, 37° 58′ N longitude-34° 41′ E latitude) (Fig. 1). The region is characterized by a semi-arid climate, with an average annual precipitation of 348.8 mm, a mean temperature of 11.1 °C, and average relative humidity of 55%.

## Tick Collection and Identification

Ticks were collected from sheep and goats during clinical examination and blood sampling. Specimens were gently removed using blunt-tipped forceps, placed individually into labeled tubes with 70% ethanol, and transported to the laboratory. Adult ticks were morphologically identified to species level under a stereomicroscope using standard taxonomic keys [8].

## Molecular Detection of *Anaplasma* spp.

Genomic DNA was extracted from the thawed blood with a GF-1 Blood DNA Extraction Kit (Vivantis Technologies Sdn. Bhd. Revongen Corporation Center, Malaysia) according to manufacturer’s instructions. The DNA concentration (ng/μL) and purity (A260nm/A280nm) of each sample was determined using a nanodrop spectrophotometer. To determine the presence and frequency of *A. bovis* (*16S rRNA*) [9], *A. capra* (*16S rRNA* and *gltA*) [5, 10], *A. ovis* (*groEL*) [11], *A. platys*-like (*groEL*) [12] and *A. phagocytophilum* (*msp4*) [13] in small ruminants, species specific PCRs were set up using different primer sets. The PCR reactions were performed in PCR Sprint (Sensoquest, Germany) as previously described [14].

## Statistical Analysis

The descriptive statistics were analyzed by SAS program. The means and confidence internals were calculated using relevant SAS procedures. The presence of *Anaplasma* species were grouped by the age of animals as well as sampling seasons, i.e., groups of April, May, June and July. Then, these groups were subjected to significance test by chi-square (χ2) homogeneity tests. In these tests, *p* < 0.05 were considered to be statistically significant.

## Results

### Molecular Detection and Overall Prevalence of *Anaplasma* spp.

Of the 690 samples examined by PCR, 437 (%63.3; CI 59.1–66.9) were infected with *Anaplasma* spp. As a result of the species specific PCR, only *A. ovis* positivity was detected sheep and goat, %69.4 (CI 65.3–73.3) and %44.7 (CI 37.1–52.5) respectively. No positivity was detected in type specific PCRs of different gene regions specific to *A. phagocytophylium* (Msp4), *A. bovis* (16S rRNA), *A. capra* (16S rRNA and gltA) and *A. platys*-like (groEL).

## Seasonal Distribution of *Anaplasma ovis*

**Fig. 2:**
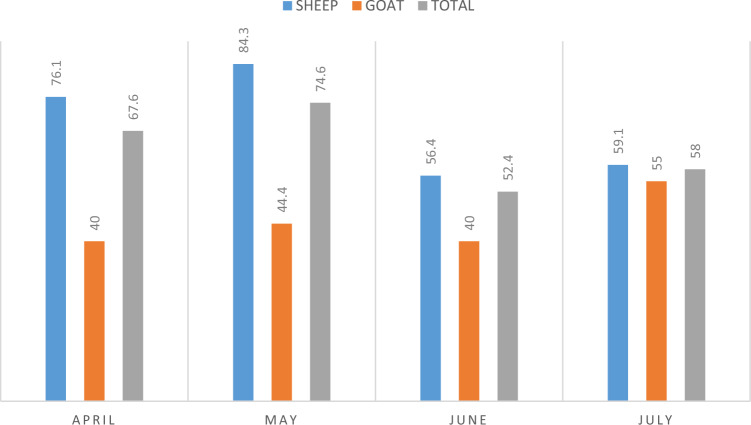
Distribution of *Anaplasma ovis* positivity according to the months in which the samples were collected

The monthly distribution of *A. ovis* positivity is shown in Table 1 and Fig. 2. *Anaplasma ovis* positivity was observed to be the highest in May (74.6%) and the lowest in June (52.4%). It was observed that there was the highest positivity for sheep in May (84.3%) and for goats in July (55%). The difference between the months was not statistically significant (*p* > 0.05).

**Table 1:** Distribution of the species-specific PCR results by months

Months	Host	Number of samples	*Anaplasma* spp.	*A. ovis*	*A. phagocytophylium*	*A. bovis*		*A. capra*		*A. platys-*like	
April	Sheep	130	99 (%76.1; 67.9–83.2)	99; (%76.1; 67.9–83.2)	–	–		–		–	
	Goat	40	16 (%40; 24.9–56.7)	16 (%40; 24.9–56.7)	–	–		–		–	
	∑	170	115 (%67.6; 60–74.6)	115 (%67.6; 60–74.6)	–	–		–		–	
May	Sheep	140	118 (%84.3; 77.2–89.9)	118 (%84.3; 77.2–89.9)	–	–		–		–	
	Goat	45	20 (%44.4; 29.6–60)	20 (%44.4; 29.6–60)	–	–		–		–	
	∑	185	138 (74.6; 67.7–80.7)	138 (74.6; 67.7–80.7)	–	–		–		–	
June	Sheep	140	79 (%56.4; 47.8–64.8)	79 (%56.4; 47.8–64.8)	–	–		–		–	
	Goat	45	18 (%40; 25.7–55.7)	18 (%40; 25.7–55.7)	–	–		–		–	
	∑	185	97 (%52,4; 45–59.8)	97 (%52.4; 45–59.8)	–	–		–		–	
July	Sheep	110	65 (%59,1; 49.3–68.4)	65 (%59.1; 49.3–68.4)	–	–		–		–	
	Goat	40	22 (%55; 38.5–70.7)	22 (%55; 38.5–70.7)	–	–		–		–	
	∑	150	87 (%58; 49.7–66)	87 (%58; 49.7–66)	–	–		–		–	
Total	Sheep	520	361 (%69.4; 65.3–73.3)	361 (%69.4; 65.3–73.3)	–	–		–		–	
	Goat	170	76 (%44.7; 37.1–52.5)	76 (44.7; 37.1–52.5)	–	–		–		–	
	∑	690	437 (%63.3; 59.1–66.9)	437 (%63.3; 59.1–66.9)	–	–		–		–

## Distribution of *Anaplasma ovis* Infections by Age

**Fig. 3:**
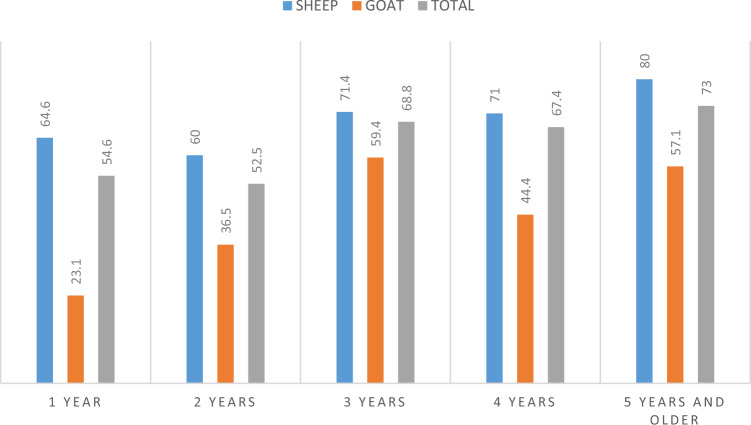
Distribution of *Anaplasma ovis* positivity according to the ages

According to Table 2 and Fig. 3, *A. ovis* positivity was determined in each age group. It was observed that the highest positivity was at the age of 5 years and over (73%), and the least positivity was in the 2 ages group (52.5%). The highest positivity in sheep was 5 years old and over (80%), and the highest positivity in goats was determined in the 3 ages group (59.4). The difference between the age groups was not statistically significant (*p* > 0.05).

**Table 2:** Distribution of *Anaplasma ovis* in sheep and goats by age

Age	Host	n	*A. ovis* (%; 95% CI)
1 year	Sheep	82	53 (64.6; 53.3–74.9)
	Goat	26	6 (23.1; 9.0–43.6)
	∑	108	59 (54.6; 44.8–64.2)
2 years	Sheep	110	66 (60; 50.2–69.2)
	Goat	52	19 (36.5; 23.6–51.0)
	∑	162	85 (52.5; 44.5–60.3)
3 years	Sheep	119	85 (71.4; 62.4–79.3)
	Goat	32	19 (59.4; 40.6–76.3)
	∑	151	104 (68.8; 60.8–76.1)
4 years	Sheep	114	81 (71; 61.8–79.1)
	Goat	18	8 (44.4; 21.5–69.2)
	∑	132	89 (67.4; 58.7–75.3)
5 years and older	Sheep	95	76 (80; 70.5–87.5)
	Goat	42	24 (57.1; 41–72.3)
	∑	137	100 (73; 67.5–82.8)
Total	Sheep	520	361 (69.4; 65.3–73.3)
	Goat	170	76 (44.7; 37.1–52.5)
	∑	690	437 (63.3; 59.1–66.9)

## Species Distribution of Ticks Collected from Small Ruminants

In our previous study using the same material [15], a total of 1361 (579♀, 782♂) ticks were collected from sheep and goats with tick infestation, 383 (28.1%) of them were *R. bursa*, 607 (44.6%) were *R. turanicus*, 7 (0.5%) were *Hy. marginatum*, 247 (18.1%) of *Hy. excavatum*, 23 (1.7%) of *Hy. anatolicum*, 21 (1.5%) *Hae. parva*, 7 (0.5%) *Hae. punctata*, 40 (2.9%) *Hae. sulcata* and 26 (1.9%) *D. marginatus* were identified.

## Discussion and Conclusion

The current study detected a high prevalence of *A. ovis* infection among sheep and goats in Niğde province located in Central Türkiye, but tests did not provide any evidence for other *Anaplasma* species, including the zoonotic *A. capra* and *A. phagocytophilum*. Previous studies conducted in Türkiye have reported varying prevalence rates of *A. ovis* (31.4–67%) and *A. phagocytophilum* (2.4–66.7%) in small ruminants [16–20]. Moreover, *A. ovis* has been recorded in different countries with various ecological conditions such as Iraq (66.6%), Sudan (41.6%), Portugal (84.2%) [16], Tunisia (70.1%) [21], Hungary (72.7%) [22], Italy (87%) [23], China (14.3%) [24] and Slovakia (22.6%) [25], showing its wide geographical distribution and adaptability.

One explanation for the detection of *A. ovis* only in this study is based on the ecology of the area and the distribution of tick vectors. The climate of Niğde province is arid to semi-arid, generally being unsuitable for the persistence of *Ixodes ricinus*, the main vector of *A. phagocytophilum* [26]. Although the specific vector of *A. capra* is yet to be identified, there have been reports implicative of some relation with *Haemaphysalis* species, particularly in East Asia [27]. The sporadic detection of *Haemaphysalis* spp. in the present study may indicate a limited role for these ticks in the local transmission dynamics of *A. capra*. Additionally, the finding of *R. bursa* and *R. turanicus* species, which are the possible vector of the *A. ovis*, agrees with the high infection rates recorded and reflects the presence of active circulation and transmission in the small ruminant population [28–30].

The age-related distribution of *A. ovis* infection in this study showed varying prevalence across groups, with the highest rate observed in animals aged five years and older. However, the difference among age groups was not statistically significant. While the absence of a significant association suggests that age may not be a major determinant under the current conditions, higher prevalence in older animals has been reported in other studies and is often attributed to prolonged environmental exposure during grazing and trade activities [31, 32]. Conversely, some investigations have reported no age-related pattern, indicating that local management practices, vector density, and host immunity may play more influential roles [30, 33].

No clinical signs were observed in any of the animals sampled, but the high prevalence of infection indicates a generalized subclinical circulation of *A. ovis*. These infections may lead to decreased performance, lack of resistance against secondary infections and may become immunosuppressive, especially in stress and/or co-infection situations [34]. Although *A. ovis* has traditionally been regarded as non-zoonotic, a single report from Cyprus documented an *A. ovis*-like variant detected in a human patient, raising questions about its potential zoonotic capacity [35]. While further studies are required to confirm pathogenicity in humans, this finding highlights the need to reassess the public health implications of *A. ovis* in endemic areas.

This study reports that *A. ovis* is the most prevalent *Anaplasma* species of small ruminants in Niğde province, Central Türkiye, with a high prevalence in total and subclinically circulating. The inability to detect zoonotic strains such as *A. capra* and *A. phagocytophilum*, could be due to local ecological barriers and low vector competence. *Anaplasma ovis* has conventionally been perceived as non-zoonotic; however, a human case was reported which indicates its potential public health concern. These findings emphasize the need for increased awareness of anaplasmosis among farmers, veterinarians, and public health authorities, especially in endemic rural areas. Continued surveillance and molecular characterization of circulating *Anaplasma* species are essential not only for improving animal health management but also for anticipating emerging zoonotic threats.

## Data Availability

No datasets were generated or analysed during the current study.

## References

[1] Aubry P, Geale DW (2011) A review of Bovine Anaplasmosis. Transbound Emerg Dis 58:1–30. 10.1111/j.1865-1682.2010.01173.x 21040509 10.1111/j.1865-1682.2010.01173.x

[2] Atif FA (2016) Alpha proteobacteria of genus *Anaplasma* (Rickettsiales: Anaplasmataceae): epidemiology and characteristics of *Anaplasma* species related to veterinary and public health importance. Parasitology 143:659–685. 10.1017/S0031182016000238 26932580 10.1017/S0031182016000238

[3] Friedhoff KT (1997) Tick-borne diseases of sheep and goats caused by *Babesia*, *Theileria* or *Anaplasma* spp. Parassitologia 39:99–109 9530692

[4] Stuen S (2013) Tick-borne infections in small ruminants in northern Europe. Small Rumin Res 110:142–144. 10.1016/j.smallrumres.2012.11.022

[5] Li H, Zheng YC, Ma L, Jia N, Jiang BG, Jiang RR, Huo QB, Wang YW, Liu HB, Chu YL, Song YD, Yao NN, Sun T, Zeng FY, Dumler JS, Jiang JF, Cao WC (2015) Human infection with a novel tick-borne *Anaplasma* species in China: a surveillance study. Lancet Infection Disease 15:663–670. 10.1016/S1473-3099(15)70051-4 10.1016/S1473-3099(15)70051-4 25833289

[6] Ben Said M, Belkahia H, Karaoud M, Bousrih M, Yahiaoui M, Daaloul-Jedidi M, Messadi L (2015) First molecular survey of *Anaplasma bovis* in small ruminants from Tunisia. Vet Microbiol 179:322–326. 10.1016/j.vetmic.2015.05.022 26088935 10.1016/j.vetmic.2015.05.022

[7] Liu Z, Ma M, Wang Z, Wang J, Peng Y, Li Y, Guan G, Luo J, Yin H (2012) Molecular survey and genetic identification of *Anaplasma* species in goats from central and southern China. Appl Environ Microbiol 78:464–470. 10.1128/AEM.06848-11 22057867 10.1128/AEM.06848-11 PMC3255723

[8] Estrada-Pena A, Bouattour A, Camicas JL, Walker AR (2004) Ticks of domestic animals in the Mediterranean region: a guide to identification of species. Publised by University of Zaragoza, Spain

[9] Kawahara M, Rikihisa Y, Lin Q, Isogai E, Tahara K, Itagaki A, Hiramitsu Y, Tajima T (2006) Novel genetic variants of *Anaplasma phagocytophilum*, *Anaplasma bovis*, *Anaplasma centrale*, and a novel *Ehrlichia* sp. in wild deer and ticks on two major islands in Japan. Appl Environ Microbiol 72:1102–1109. 10.1128/AEM.72.2.1102-1109.2006 16461655 10.1128/AEM.72.2.1102-1109.2006 PMC1392898

[10] Yang JF, Liu ZJ, Niu QL, Liu JL, Han R, Liu GY, Shi YX, Luo JX, Yin H (2016) Molecular survey and characterization of a novel *Anaplasma* species closely related to *Anaplasma capra* in ticks, northwestern China. Parasite Vectors 9:603. 10.1186/s13071-016-1886-6 10.1186/s13071-016-1886-6 PMC5123347 27884197

[11] Haigh JC, Gerwing V, Erdenebaatar J, Hill JE (2008) A novel clinical syndrome and detection of *Anaplasma ovis* in Mongolian reindeer (Rangifer tarandus). Journal of Wildlife Disease 44:569–577. 10.7589/0090-3558-44.3.569 10.7589/0090-3558-44.3.569 18689641

[12] Alberti A, Sparagano OA (2006) Molecular diagnosis of granulocytic anaplasmosis and infectious cyclic thrombocytopenia by PCR-RFLP. Ann N Y Acad Sci 1081:371–378. 10.1196/annals.1373.055 17135540 10.1196/annals.1373.055

[13] De la Fuente J, Massung RF, Wong SJ, Chu FK, Lutz H, Meli M, von Loewenich FD, Grzeszczuk A, Torina A, Caracappa S, Mangold AJ, Naranjo V, Stuen S, Kocan KM (2005) Sequence analysis of the msp4 gene of *Anaplasma phagocytophilum* strains. J Clin Microbiol 43:1309–1317. 10.1128/JCM.43.3.1309-1317.2005 15750101 10.1128/JCM.43.3.1309-1317.2005 PMC1081214

[14] Ozubek S, Aktas M (2017) Molecular evidence of a new *Babesia* sp. in goats. Veterinary Parasitology 15: 233:1–8. 10.1016/j.vetpar.2016.11.016 10.1016/j.vetpar.2016.11.016 28043378

[15] Karatepe B, Özübek S, Karatepe M, Aktaş M (2019) Detection of *Theileria* and *Babesia* species in sheep and goats by microscopy and molecular methods in Nigde province, Turkey. Revue de Medecine Veterinaire 170:136–143

[16] Renneker S, Abdo J, Salih DEA, Karagenc T, Bilgic H, Torina A, Oliva AG, Campos J, Kullmann B, Ahmed J, Seitzer U (2013) Can *Anaplasma* ovis in small ruminants be neglected any Longer? Transbound Emerg Dis 60:105–112. 10.1111/tbed.12149 24589109 10.1111/tbed.12149

[17] Altay K, Dumanlı N, Aktas M, Ozubek S (2014) Survey of *Anaplasma* infections in small ruminants from East Part of Turkey. Kafkas Universitesi Veteriner Fakültesi Dergisi 20:1–4. 10.9775/kvfd.2013.9189

[18] Öter K, Çetinkaya H, Vuruşaner C, Toparlak M, Ergünay K (2016) Molecular detection and typing of *Anaplasma* species in small ruminants in Thrace Region of Turkey. Kafkas Universitesi Veteriner Fakültesi Dergisi 22:133–138. 10.9775/kvfd.2015.14075

[19] Bilgic HB, Bakirci S, Kose O, Unlu AH, Hacilarlioglu S, Eren H, Weir W, Karagenc T (2017) Prevalence of tick-borne haemoparasites in small ruminants in Turkey and diagnostic sensitivity of single-PCR and RLB. Parasites Vectors 10:211. 10.1186/s13071-017-2151-3 28449722 10.1186/s13071-017-2151-3 PMC5408456

[20] Benedicto B, Ceylan O, Moumouni PFA, Lee SH, Tumwebaze MA, Li J, Galon EM, Liu M, Li Y, Ji S, Ringo A, Rizk M, Sevinc F, Xuan X (2020) Molecular detection and assessment of risk factors for tick-borne diseases in sheep and goats from Turkey. Acta Parasitol 65:723–732. 10.2478/s11686-020-00207-0 32378157 10.2478/s11686-020-00207-0

[21] Belkahia H, Ben Said M, Sayahi L, Alberti A, Messadi L (2015) Detection of novel strains genetically related to *Anaplasma platys* in tunisian one-humped camels [*Camelus dromedarius*]. J Infect Dev Ctries 9:117–1125. 10.3855/jidc.6950 10.3855/jidc.6950 26517487

[22] Hornok S, Elek V, de la Fuente J, Farkas NVR, Majoros G, Földvári G, (2007) First serological and molecular evidence on the endemicity of *Anaplasma ovis* and *A. marginale* in Hungary. Vet Microbiol 122:316–322. 10.1016/j.vetmic.2007.01.024 10.1016/j.vetmic.2007.01.024 17336001

[23] De la Fuente J, Torina A, Caracappa S, Tumino G, Furlá R, Almazán C, Kocan KM (2005) Serologic and molecular characterization of *Anaplasma* species infection in farm animals and ticks from Sicily. Vet Parasitol 133:357–362. 10.1016/j.vetpar.2005.05.063 16043300 10.1016/j.vetpar.2005.05.063

[24] Han R, Yang J, Liu Z, Gao S, Niu Q, Hassan MA, Luo J, Yin H (2017) Characterization of *Anaplasma ovis* strains using the major surface protein 1a repeat sequences. Parasit Vectors 10:447. 10.1186/s13071-017-2363-6 28962625 10.1186/s13071-017-2363-6 PMC5622584

[25] Derdáková M, Stefančíková A, Spitalská E, Tarageľová V, Košťálová T, Hrkľová G, Kybicová K, Schánilec P, Majláthová V, Várady M, Peťko B (2011) Emergence and genetic variability of *Anaplasma* species in small ruminants and ticks from Central Europe. Vet Microbiol 153:293–298. 10.1016/j.vetmic.2011.05.044 21684091 10.1016/j.vetmic.2011.05.044

[26] Černý J, Lynn G, Hrnková J, Golovchenko M, Rudenko N, Grubhoffer L (2020) Management options for Ixodes ricinus-associated pathogens: a review of prevention strategies. Int J Environ Res Public Health 17:1830. 10.3390/ijerph17061830 32178257 10.3390/ijerph17061830 PMC7143654

[27] Altay K, Erol U, Sahin OF (2024) *Anaplasma capra*: a new emerging tick-borne zoonotic pathogen. Vet Res Commun 48:1329–1340. 10.1007/s11259-024-10337-9 38424380 10.1007/s11259-024-10337-9 PMC11147849

[28] Aktas M, Altay K, Dumanli N, Kalkan A (2009) Molecular detection and identification of *Ehrlichia* and *Anaplasma* species in ixodid ticks. Parasitol Res 104:1243–1248. 10.1007/s00436-009-1377-1 19247690 10.1007/s00436-009-1377-1

[29] Aktas M (2014) A survey of ixodid tick species and molecular identification of tick-borne pathogens. Vet Parasitol 200:276–283. 10.1016/j.vetpar.2013.12.008 24424312 10.1016/j.vetpar.2013.12.008

[30] Aktas M, Özübek S (2018) *Anaplasma ovis* genetic diversity detected by major surface protein 1a and its prevalence in small ruminants. Vet Microbiol 217:13–17. 10.1016/j.vetmic.2018.02.026 29615245 10.1016/j.vetmic.2018.02.026

[31] Belkahia H, Ben Said M, El Hamdi S, Yahiaoui M, Gharbi M, Daaloul-Jedidi M, Mhadhbi M, Jedidi M, Darghouth MA, Klabi I, Zribi L, Messadi L (2014) First molecular identification and genetic characterization of *Anaplasma ovis* in sheep from Tunisia. Small Rumin Res 121:404–410. 10.1016/j.smallrumres.2014.07.009

[32] Yousefi A, Rahbari S, Shayan P, Sadeghi-dehkordi Z, Bahonar A (2017) Molecular detection of *Anaplasma marginale* and *Anaplasma ovis* in sheep and goat in West Highland pasture of Iran. Asian Pac J Trop Biomed 7:455–459. 10.1016/j.apjtb.2017.01.017

[33] Naeem M, Amaro-Estrada I, Taqadus A, Swelum AA, Alqhtani AH, Asif M, Sajid M, Khan AU, Tariq A, Anjum S, Khan A, Iqbal F (2023) Molecular prevalence and associated risk factors of *Anaplasma ovis* in Pakistani sheep. Front Veterinary Sci 10:1096418. 10.3389/fvets.2023.1096418 10.3389/fvets.2023.1096418 PMC10095557 37065244

[34] Jiménez C, Benito A, Arnal JL, Ortín A, Gómez M, López A, Villanueva-Saz S, Lacasta D (2019) *Anaplasma ovis* in sheep: experimental infection, vertical transmission and colostral immunity. Small Rumin Res 178:7–14. 10.1016/j.smallrumres.2019.07.003

[35] Chochlakis D, Ioannou I, Tselentis Y, Psaroulaki A (2010) Human anaplasmosis and *Anaplasma ovis* variant. Emerg Infect Dis 16:1031–1032. 10.3201/eid1606.090175 20507768 10.3201/eid1606.090175 PMC3086243

